# Management protocols and encountered complications among stroke patients admitted to stroke unit of Jimma university medical center, Southwest Ethiopia: Prospective observational study

**DOI:** 10.1016/j.amsu.2019.11.003

**Published:** 2019-11-15

**Authors:** Ginenus Fekadu, Legese Chelkeba, Tsegaye Melaku, Busha Gamachu, Mohammed Gebre, Firomsa Bekele, Getahun Fetensa

**Affiliations:** a Department of Pharmacy, Institute of Health Science, Wollega University, Nekemte, Ethiopia; b School of Pharmacy, Institute of Health, Jimma University, Jimma, Ethiopia; c Department of Pharmacy, College of Health Science, Mettu University, Mettu, Ethiopia; d Department of Nursing, Institute of Health Science, Wollega University, Nekemte, Ethiopia

**Keywords:** Stroke, Complications, Management, Treatment, Jimma, Ethiopia, AHA/ASA, The American Heart Association/ American Stroke Association, HS, Hemorrhagic stroke, IS, Ischemic stroke, JUMC, Jimma university medical center, r-tPA, Recombinant tissue plasminogen activator, SU, Stroke unit, WHO, World Health Organization

## Abstract

**Objectives:**

The number of stroke patients receiving recombinant tissue plasminogen activator (r-tPA) in developing world is extremely low. Pre-hospital delay, financial constraints and lack of infrastructure are the main barriers. Additionally, various medical, neurological and psychological complications are allied to stroke patients after the acute attack. Hence, the study was aimed to identify management protocols and encountered complications among stroke patients admitted to stroke unit of Jimma university medical center (JUMC).

**Patients and methods:**

Prospective observational study was conducted at stroke unit of JUMC from March 10 to July 10, 2017. All eligible consecutive stroke patients of ≥18 years were included. Data was entered to Epi data version 3.1 and analyzed using SPSS version 20.

**Results:**

A total of 116 eligible stroke patients were followed with mean age of 55.14 ± 14.04 years and males comprised of 62.9%. Using world health organization (WHO) criteria, 60 (51.7%) patients had ischemic while 56 (48.3%) had hemorrhagic stroke. During admission, 93 (80.2%) patients had developed at least one complication. The most complication was brain edema (increased intracranial pressure) detected in about one third of the patients (30.2%) followed by urinary incontinence (28.4%) and aspiration pneumonia (19.8%). Almost half of the patients (52.5%) had at least one past medication history. During hospitalization, 111(95.7%) of patients had received at least one medication and the median start time of the medications after hospital arrival was 15 h. The most common medication given for the patients during hospitalization were antiplatelets and statins for 63 (54.3%) patients. During discharge, about 78 (67.2%) patients had received medications and antihypertensives were the prominent medications prescribed for half of the discharged patients.

**Conclusion:**

Both neurologic and medical complications were common in majority of stroke patients. But the management protocol for stroke patients was sub-optimal and lagging behind the recommended guidelines due to lack of skilled personnel, appropriate treatment and diagnostic agents. The clinical team involved in the care of stroke patients should make preparations to take preventive measures that will save a lot of lives.

## Highlights

• Using WHO criteria, there were almost equal prevalence's of ischemic and hemorrhagic stroke subtypes.
• The most complication developed among stroke patients were brain edema, urinary incontinence and aspiration pneumonia.
• More than half of the patients had at least one past medication history; commonly antihypertensive medications.
• The most commonly prescribed medications for the patients during hospitalization were antiplatelets and statins.
• During discharge about two third of patients had received medications.

## 1 Introduction

As per world health organization (WHO) criteria, stroke is a rapidly developing clinical signs of focal or global disturbance of cerebral function, with symptoms lasting ≥24 h leading to death, with no apparent cause other than vascular origin [[1], [2], [3]]. As heart disease and stroke statistics of 2016 report from American heart association (AHA), stroke was the second-leading global cause of death behind heart disease in 2013, accounting for 11.8% of total deaths [4]. Although stroke incidence in high-income countries (HICs) decreased over the past four decades, the burden is increasing dramatically (more than double) in low- and middle-income countries (LMICs [3,[5], [6], [7], [8]]. Globally, 70% of strokes and 87% of both stroke-related deaths and disability-adjusted life years occur in LMICs [9]. Stroke is generally regarded as a spiritual disease in Africa and there is an enormous stroke burden and mortality in sub Saharan Africa (SSA) [10].

There were differences in the prevalence of major risk factors among the stroke subtypes, demonstrating that knowledge of pathophysiology is essential for the proper management of these patients [11]. The American heart/stroke association (AHA/ASA) recommends tissue plasminogen activator (tPA), antiplatelet (aspirin, clopidogrel), anticoagulants (heparin, warfarin), antihypertensive and lipid-lowering agents for the treatment if ischemic stroke whereas osmotherapy, neuromuscular relaxants, neuroprotection and neuro-restoration therapy, reperfusion therapy and calcium channel blockers for hemorrhagic stroke [12,13]. In resource-limited settings without access to neuroimaging, administration of aspirin to all patients presenting with acute stroke of undetermined etiology could result in improved outcomes at hospital discharge [14].

The proportion of hemorrhagic stroke (HS) is higher and greater case fatality in African and other LMICs than in high income countries [[15], [16], [17]]. Although this disparity is often described to racial or genetic factors, it may actually be due to differences in risk factor burden, hospital admission bias, population pyramid, socio-economic, environmental and dietary across these population [16]. Additionally, almost equal prevalence of stroke subtypes were detected by Nkoku et al. in Nigeria, Tirschwell et al. in vietnam and Deresse et al. in Ethiopia [[18], [19], [20]]. Additionally, hemorrhagic stroke was more diagnosed than ischemic stroke in other studies [21,22].

Current guidelines for the management of acute stroke recommend all patients admitted to hospital with suspected acute stroke should receive brain imaging evaluation like computed tomography (CT) scanner on arrival to hospital to make decisions about acute management [9,13,23]. There have been many advances in management of cerebrovascular diseases. However, stroke is still one of the leading causes of disabilities and mortality worldwide with significant socio-economic burden [24]. Despite the best care, patients remain at high risk of medical complications [25]. Case-fatality was high compared with western cohorts and rational treatment, in the absence of head imaging for initial assessment requires adapted protocols [26]. Careful prevention, early diagnosis and treatment of complications after stroke are important to facilitate recovery [27].

Although surveillance, screening, and accurate diagnosis are important for stroke prevention, LMICs face challenges due to lack of resources, awareness and technical capacity [6]. Computed tomography (CT) scan is used to distinguish between stroke subtypes. Yet the majority of strokes worldwide (around 70% of approximately 17 million per annum) occur in LMICs with limited access to CT scan prior to initiation of intravenous (IV) tPA in most patients [9,13]. In low-resource settings, CT scanners are either unavailable or unaffordable, forcing clinicians to make difficult clinical decisions, such as whether to anticoagulate patients or not, and to what level to control their blood pressure without a means of distinguishing between ischemic and hemorrhagic stroke [3,28].

One systematic review by Pandian et al. revealed that the burden of stroke in LMICs is large and increasing as very few patients were thrombolysed. Adherence to secondary preventive drugs is also affected by limited availability and affordability, emphasizing the importance of primary prevention [29]. Treatment for acute stroke in developing countries is generally symptomatic; thrombolytic and neuroprotective drugs are the exception rather than the rule [30]. Long-term socio-economic and cultural activities were also affected in most patients and drug compliance is also poor [26]. Most survivors improved functionally but were left with significant disability [10]. Virtually all of the excess morbidity and mortality that occur as a consequence of stroke and other neurological disorders in developing counties result from scarcities of resources [31]. Furthermore, as most guidelines are based on datas from developed countries, uncertainty remains regarding best management of stroke of unknown type in LMICs [23].

The number of stroke patients receiving r-tPA in the developing world is extremely low. Pre-hospital delay, financial constraints and lack of infrastructure are the main barriers of thrombolytic therapy in developing countries [32]. This is because of the fact that thrombolytic therapy has been proven to be beneficial if drugs are administered only within 4.5 h after the onset of an ischemic stroke [13,22,31]. Trials have also confirmed that thrombectomy beyond 6 h and up to 24 h since stroke onset substantially benefits patients with large vessel occlusion and salvageable brain tissue [33].

Various medical, neurological and psychological complications are allied to stroke after the acute attack. This medical and neurological complications are an ongoing and predictable part of inpatient rehabilitation after stroke and are a major cause of morbidity and mortality as well as influence functional outcome [23,[34], [35], [36]]. This complications can also hinder functional recovery, can extend the hospital length of stay (LOS), worsen stroke outcomes and increase cost of care. In addition, some patients need to be transferred back to the acute care setting, which interrupts the inpatient rehabilitation therapy and further increases the overall cost of stroke management [25,27]. Even though the complications adversely impact outcome, some of these complications could be prevented or, when this is not possible, early detection and proper management could be effective in reducing the adverse effects [23,35].

Stroke is currently observed to be one of the commonest reasons of admission in many hospitals and becoming an increasingly serious public health issue in Ethiopia [37,38]. In Ethiopia significant delays during pre-hospital or in-hospital phases of care augmented by insufficient management also making prognosis of stoke patients very poor [22,39]. As patients usually present late and the standard of care is poor, the in hospital mortality is expected to be higher [38,40]. Patients with stroke are often poorly managed and discharged from hospital without receiving adequate rehabilitation services. This has a series implication in terms of saving the life of patients especially with hemorrhagic stokes which are characterized by sever neurologic presentation [22]. Under-diagnosing of hypertension and other risk factors as well as delayed presentation to the hospital are the major challenges to be addressed [37]. Although stroke has been consistently reported as one of the three leading causes of morbidity and mortality in the past years in Ethiopia, there is a paucity of data regarding complications and effective treatment; limiting the formulation of an appropriate response [37,41].

## 2 Patients and methods

### 2.1 Study setting, study design and eligibility criteria

The study was conducted at stroke unit (SU) of Jimma university medical center (JUMC), a tertiary hospital found in Jimma city, south-west Ethiopia, which is 352 kilometers from Addis Ababa. It is the main referral center for neurology patients in south west Ethiopia. SU of JUMC was established with assistance of project from United Kingdom aid direct (UKAID) and tropical health and education trust (THET) with help of Southampton hospital in 2015. Prospective observational study design was conducted for consecutive 4 months from March 10 to July 10, 2017. Since this data was part of study previously described by Fekadu et al. [28,39,40,42], the study participants in this finding share similarity with previously published articles of the same study project. The work has been reported in line with the strengthening the reporting of cohort studies in surgery (STROCSS) criteria [43].

All 116 patients ≥18 years with stroke and admitted to SU of JUMC within the study period were included. Patients or guardians not willing to give an informed consent, those who died before evaluation as well as after further evaluation if initial diagnosis of stroke was later changed to other case (ruled out stroke) as long as the patient was in the SU were excluded from the study.

### 2.2 Data collection tool and procedure

Data collection was carried out by two Bachelor of Science (BSc) nurses and one internal medicine resident with training on the contents of data collection tool. Data collectors collect all relevant data from patients chart and interviewing the patients/care givers using a prepared data extraction form. The needed history used for the study was taken from the patient and/or relatives in the language they understood. Controversial data's like laboratory interpretation, drug therapy problems and unclear data's on patients chart was discussed with physicians working in the stroke unit of JUMC during study period. Data collection tool included baseline socio-demographic information's, encountered complications and management approaches of stroke patients.

### 2.3 Data processing and analysis

All completed data collection forms was checked and examined for their completeness, consistency, clarity and accuracy. Checked data was cleaned and entered to Epidata version 3.1 and analyzed using statistical package for the social sciences (SPSS) version 20. Descriptive statistics were calculated to describe the sociodemographic characteristics, encountered complications, management protocols and time to start of medications. Chi-square (χ2) test was used to test the significance of associations between categorical variables and Kolmogorov–Smirnov test was used to assess the distribution of variables. Predictor with probability value less than 0.05 was considered statistically significant.

### 2.4 Ethics approval and consent to participate

Ethical clearance was obtained from the Institutional Review Board (IRB) of Institute of health, Jimma University. Verbal consent was obtained from responsible bodies of the hospital and stroke unit prior to interviewing and reviewing patient data. At hospital, from patients written consent was obtained and all patients got the right to opt out of the research. The data from the case records and interview was handled with strong confidentiality. This confidentiality and privacy of patients was assured throughout by removing identifiers from data collection tools using different codes. The study was registered researchregistry.com with unique reference number of “researchregistry5112”.

### 2.5 The standard protocols for stroke diagnosis and management

The goal of treating stroke is to reduce the ongoing neurologic injury and decrease mortality and long-term disability. It also targeted to prevent complications secondary to immobility and neurologic dysfunction, and preventing stroke recurrence. Minimizing the volume of brain that is irreversibly damaged and handicap of the patients through rehabilitation were other prioritized areas of the treatment [25,31]. Ethiopian hospitals haven't well-established stroke units or stroke teams, and no official national protocol for acute stroke management during the study period. Even guideline by Ethiopian Medicine, Food and Healthcare Administration and Control Authority (EMFHACA) for general hospitals doesn't put any recommendation and protocol regarding stroke management in Ethiopia [44]. But globally there are numerous protocols for stroke management and rehabilitation. Management of stroke includes primary prevention, active management of the acute stroke phase, secondary prevention and rehabilitation. The rehabilitation starts by preventing, recognizing and managing co-morbid illness and medical complications [31]. Maintaining a healthy lifestyle such as no tobacco use, healthful diet, and physical activity are also important strategies for both primary and secondary prevention of stroke [6]. During acute inpatient care, stroke patients should undergo appropriate investigations to determine stroke mechanism and guide stroke prevention and management decisions [25].

Patients with acute stroke (onset within last 72 h or altered consciousness due to stroke) should be admitted to hospital for initial care and assessment. All levels of stroke care facilities should have the capability of performing or access to either a cranial computed tomography (CT scan) or magnetic resonance imaging (MRI) scan within 30 min of the order being written with experienced physicians or a radiologist to interpret the imaging reports. Patients should also receive clinical diagnosis, CT brain scan, specialist evaluation by a multidisciplinary team and protocols should be made available for rt-PA use in acute stroke [45,46].

As per world health organization (WHO), lowering systolic blood pressure is recommended for patients with acute intracerebral hemorrhage who present with elevated blood pressure (BP) to below 140 mmHg. After acute ischemic stroke, blood pressure is often allowed to autoregulate unless thrombolytic therapy is administered. In cases of acute ischemic stroke in which intravenous t-PA activator is administered, blood pressure is subsequently maintained below 180/105 mmHg. when CT is not available to distinguish between hemorrhagic and ischemic stroke, it may be reasonable to consider lowering systolic blood pressure to below 180 mmHg for all patients with acute stroke of unknown etiology. When CT is not available, IV t-PA cannot be safely administered, and aspirin is generally the only antithrombotic agent available [9].

AHA/ASA guideline of 2018 recommends administration of aspirin for patients with acute ischemic stroke (AIS) within 24–48 h after onset. For those treated with IV alteplase, aspirin administration is generally delayed until 24 h later. Urgent anticoagulation (e.g., heparin drip) for most stroke patients is not indicated. Patients who have elevated BP and are otherwise eligible for treatment with IV alteplase should have their BP < 185/110 mm Hg before IV fibrinolytic therapy is initiated. IV alteplase (0.9 mg/kg, maximum dose 90 mg over 60 min with initial 10% of dose given as bolus over 1 min) is recommended for selected patients who may be treated within 3–4.5 h of ischemic stroke symptom onset [13].

## 3 Result

Using WHO criteria, from 116 patients, 60 (51.7%) patients had ischemic stroke while 56 (48.3%) had hemorrhagic stroke.

### 3.1 Socio-demographic characteristics of the patients

The mean age of the patients was 55.14 ± 14.04 years. Males comprised of 73 (62.9%) with male: female ratio of 1.70:1. Majority of the participants, (42.2%) had informal education (able to read and write) followed by no basic education (36.2%). More than one third (37.9%) of them were farmers as their recent occupation. As per to this finding, none of the socio-demographic factors had association with stroke based on chi-square test (P > 0.05) [Table 1].

**Table 1:** Socio-demographic characteristics among adult stroke patients admitted to stroke unit of JUMC from March 10 to July 10, 2017.

Socio-demographic factors		Total patients (n = 116)	Ischemic stroke (n = 60)	Hemorrhagic stroke (n = 56)	P value
Age (years)	Mean ± SD	55.14 ± 14.04	57.38 ± 14.59	52.73 ± 13.14	0.187
Sex	Male	73 (62.9%)	38 (63.3%	35 (62.5%	0.926
	Female	43 (37.1%)	22 (36.7%)	21(37.5%)	
Residence	Rural	84 (72.4%)	43 (71.7%)	41 (73.2%)	0.852
	Urban	32 (27.6%	17 (28.3%)	15 (26.8%)	
Marital status	Married	104 (89.7%)	54 (90.0%)	50 (89.3%)	0.575
	Widow	11 (9.5%)	5 (8.3%)	6 (10.7%)	
	Divorced	1 (0.9%)	1 (1.7%)	0 (0%)	
Religion	Muslim	71 (61.2%)	40 (66.7%)	31 (55.4%	0.418
	Orthodox	35(30.2%)	15 (25.0%)	20 (35.7%)	
	Protestant	9 (7.8%)	4 (6.7%)	5 (8.9%)	
	Traditional belief	1(0.9%)	1 (1.7%)	–	
Education status	No basic education	42 (36.2%)	20 (33.3%)	22 (39.3%)	0.642
	Informal education	49 (42.2%)	28 (46.7%)	21 (37.5%)	
	Formal education	25(21.6%)	12(20%)	13(23.2%)	
Occupational status (over the last 1years)	Agriculture/farmer	44 (37.9%)	27 (45.0%)	17(30.4%)	0.264
	Homemaker/housewives	41 (35.3%	20 (33.3%)	21 (37.5%)	
	Other professions^a^	31(26.7%)	13(21.7%)	18 (32.1%)	

SD: standard deviation.

* Silte, Yem, Tigire, Nuwer

a Merchant, retired, government employee, other own business work, daily worker.

### 3.2 In hospital complications of stroke

During hospital admissions, a total of 93 (80.2%) [IS = 44 and HS = 49] patients had developed at least one complication. Neurologic complication was statistically significant in hemorrhagic stroke compared to ischemic stroke patients (P < 0.01). Neurological complications were developed among 53 (45.7%) [IS = 26.7%, HS = 66.1%] patients and medical complication developed among 82 (70.7%) [IS = 68.3%, HS = 73.2%] patients. Overall, there was no significant statistical difference in complications between hemorrhagic and ischemic stroke (P = 0.061).

Common neurologic complication developed was brain edema (increased intracranial pressure) which was detected in about one third of the patients (30.2%) followed by swallowing difficulties/dysphagia 13 (11.2%) and epileptic seizure 10 (8.6%). Brain edema was statistically significant in hemorrhagic stroke as compared to ischemic stroke (p < 0.001). Additionally, swallowing difficulties and intracranial hemorrhage were prominent in HS but other neurologic complications like epileptic seizure, recurrent stroke and delirium were prominent in ischemic stroke without statistically significant difference. Most medical complication developed was urinary incontinence 33 (28.4%) followed by aspiration pneumonia 23(19.8%) and electrolyte disturbance 20 (17.2%). Urinary incontinence, aspiration pneumonia and electrolyte disturbance were higher in HS but other medical complications were more prevalent in ischemic stroke without statistical difference [Table 2].

**Table 2:** The in hospital complications among adult stroke patients admitted to stroke unit of JUMC from March 10 to July 10, 2017.

Complications during hospital stay		Total	Ischemic stroke	Hemorrhagic stroke	P value
Neurological complications (n = 53)	Brain edema/increase ICP	35 (30.2%)	5 (8.3%)	30 (53.6%)	<0.001
	Swallowing difficulties/dysphagia	13 (11.2%)	5 (8.3%)	8 (14.3%)	0.315
	Epileptic seizure	10 (8.6%)	6 (10.0%)	4 (7.1%)	0.585
	Recurrent stroke/new stroke	3 (2.6%)	2 (3.3%)	1 (1.8%)	0.605
	Delirium	3 (2.6%)	2 (3.3%)	1 (1.8%)	0.605
	Central hyperthermia	2 (1.7%)	1 (1.7%)	1 (1.8%)	0.961
	Parenchymal hemorrhage/ICH	1 (0.9%)	0 (0%)	1 (1.8%)	–
Medical complications (n = 82)	Urinary incontinence	33 (28.4%)	15 (25.0%)	18 (32.1%)	0.395
	Aspiration pneumonia	23 (19.8%)	9 (15.0%)	14 (25.0%)	0.181
	Electrolyte disturbance	20 (17.2%)	8 (8.3%)	12 (21.4%)	0.252
	Urinary tract infection	17 (14.7%)	11 (18.3%)	6 (10.7%)	0.251
	Deep venous thrombosis (DVT)	9 (7.8%)	6 (10.0%)	3 (5.4%)	0.358
	Acute renal failure	8 (6.9%)	4 (6.7%)	4 (7.1%)	0.919
	Constipation	6 (5.2%)	5 (8.3%)	1 (1.8%)	0.148
	Other hospital acquired infections	5 (4.3%)	3 (5.0%)	2 (3.6%)	0.706
	Myocardial infarction (MI)	4 (3.4%)	3 (5.0%)	1 (1.8%)	0.364
	Heart failure	4 (3.4%)	4 (6.7%)	0 (0%)	0.999
	Atrial fibrillation	3 (2.6%)	3 (5.0%)	0 (0%)	0.999
	Bed sores/decubitus ulcer/	2 (1.7%)	1 (1.7%)	1 (1.8%)	0.961
	Pulmonary embolism (PE)	2 (1.7%)	2 (3.3%)	0 (0%)	0.999
	Lung infection (TB)	1 (0.9%)	1 (1.7%)	0 (0%)	–
	Falls	1 (0.9%)	1 (1.7%)	0 (0%)	–
	Phlebothrombosis	1 (0.9%)	0 (0%)	1 (1.8%)	–
	DKA	1 (0.9%)	1 (1.7%)	0 (0%)	–

DKA: diabetic keto acidosis, ICH: intracranial hemorrhage, ICP: intracranial pressure.

### 3.3 Approaches/protocols of stroke management

Of the total patients, 61 (52.5%) had at least one past medication history. The most pre-stroke medications were diuretics 33 (54.1%) followed by angiotensin converting enzyme inhibitors (ACEI) 19 (31.1%), calcium channel blockers 12 (19.7%) and antiplatelets 12 (19.7%). Pre-stroke medications were more common among ischemic stroke patients as compared to hemorrhagic stroke patients without statistically significant difference (P = 0.099). Beta blockers and anticoagulants were solely used by ischemic stroke patients as past medication history.

During hospitalization, 111(95.7%) patients [IS = 58 (96.0%) and HS = 53 (94.6%] had received at least one medication. The median start time of medications after hospital arrival was 15 h (ranged 0.5–276 h) for all patients, 14.5 h (0.5–276 h) and 15 h (1–168 h) for ischemic and hemorrhagic stroke patients, respectively. Less than one third of the patients (27%) received medication within 3 h of hospital arrival. Half of the patients (49.5%) started medication within 12 h, 83 (75.6%) within 24 h, 91 (81.9%) within 48 h and 19 (17.1%) started medication after 48 h of hospital arrival. No statistically significant difference in median time of starting medications between stroke subtypes (P = 0.838). None of the patients had received t-PA during hospital admission because of unavailability of the medications.

During hospitalization, the most common medications given for the patients were antiplatelet and statins 63 (54.3), followed by antibiotics 44 (37.9%) and ACEIS 41 (35.3%). Antiplatelet and statins were mainly given for ischemic stroke patients, but 7 patients with hemorrhagic stroke (6.0%) received these medications wrongly before stroke was confirmed by CT-scan suspecting clinically to be ischemic stroke.

The median start time of antiplatelets after hospital arrival was 24 h (ranged 0.5–276 h) for all patients, 24 h (0.5–276 h) and 4 h (1–24 h) for ischemic stroke and hemorrhagic stroke patients, respectively. Thirteen patients (20.6%) started antiplatelets within 3 h of hospital arrival, 26 (41.2%) within 6 h, 44 (69.8%) within 24 h and 30.2% after 24 h of hospital arrival. The antiplatelet prescribed were aspirin for 61 (96.8%) patients and aspirin plus clopidogrel for 2 (3.2%) patients.

Heparin was the most anticoagulant drug prescribed for 30 (78.9%) patients and mannitol was the prominent medication given for treatment of increased intracranial pressure for 35 (30.2%) patients. ACEI, thiazide diuretics, beta blockers, loop diuretics, potassium sparing diuretics, anticonvulsants, antiulcers, and laxatives were given more for ischemic stroke patients, but CCB, anti-pains and osmotic diuretics were given more for hemorrhagic stroke patients. Other supportive agents provided for patients during hospitalization were in-hospital rehabilitation therapy (bedside physiotherapy) for 115 (99.1%), salt free diet for 113 (97.4%) and sugar free diet for 8 (6.4%) patients.

During discharge, 78 (67.2%) patients [IS = 49 (81.7%) and HS = 29 (51.8%)] received medication. Antihypertensive drugs were the commonly prescribed medications during discharge and given for half of the discharged patients. The most drugs prescribed during discharge were ACEIs 40 (70%) followed by diuretics 22 (37.9%) and CCB 12 (20.7%) patients. At discharge antiplatelet and statins were provided for patients with ischemic stroke alone for 49 (42.2%) patients [Table 3].

**Table 3:** Approaches/protocols of stroke management among adult stroke patients admitted to stroke unit of JUMC from March 10 to July 10, 2017.

Management protocols and medications			Total patients (=116)	Ischemic stroke (n = 60)	Hemorrhagic stroke (n = 56)
Pre-stroke medication (past medication history) (n = 61)	Diuretics		33 (54.1%)	22 (61.1%)	11 (44.0%)
	ARB or ACEI		19 (31.1)	13 (36.1%)	6 (24.0%)
	Antiplatelet		12 19.7%)	10 (27.8%)	2 (8.0%)
	CCB		12 (19.7%)	5 (13.9%)	7 (28.0%)
	B-blocker		8 (13.1%)	8 (22.2%)	0 (0%)
	Statins		6 (9.8%)	5 (13.9%)	1 (4.0%)
	Anticoagulant		5 (8.2%)	5 (13.9%)	0 (0%)
	Antidiabetics		4 (6.6%)	3 (8.3%)	1 (4.0%)
	Unknown medications		7 (11.5%)	2 (5.6%)	5 (20.0%)
	Others		13 (21.3%)	9 (25.0%)	4 (16.0%)
Medication given during hospitalization (n = 111)	Antiplatelets (n = 63)	Aspirin	61 (96.8%)	54 (96.4%)	7 (100%)
		Aspirin + clopidogrel	2 (3.2%)	2 (3.6%)	0 (0%)
	Statin (Atorvastatin)		63 (54.3%)	56 (93.3%)	7 (12.5%)
	ACE inhibitors (n = 41)	Enalapril	27(65.9%)	21 (84%)	6 (37.5%)
		Captopril	13 (31.7%)	4 (16%)	9 (56.3%)
		Enalapril and captopril	1 (2.4%)	0 (0%)	1 (6.3%)
	Anticoagulants (n = 38)	Heparin	30 (78.9%)	22 (73.0%)	8 (100%)
		Enoxaparin	2 (5.3%)	2 (6.7%)	0 (0%)
		Warfarin	4 (10.5%)	4 (13.0%)	0(0%)
		Heparin and warfarin	1 (2.6%)	1(3.3%)	0 (0%)
		Enoxaparin and warfarin	1 (2.6%)	1 (3.3%)	0 (0%)
	Osmotic diuretics (mannitol)		35 (30.2%)	5 (8.3%)	30 (53.6%)
	Thiazide diuretics/hydrochlorothiazide		14 (12.1%)	7 (11.7%)	7 (12.5%)
	Calcium channel blocker (n = 10)	Amlodipine	5 (50.0%)	1 (50.0%)	4 (50.0%)
		Nifedipine	4 (10.0%)	1 (50.0%)	3 (37.5%)
		Nifedipine and amlodipine	1 (10.0%)	0 (0%)	1 (12.5%)
	Beta blockers (n = 9)	Atenolol	1 (11.1%)	0 (0%)	1 (33.3%)
		propranolol	1 (11.1%)	1 (16.7%)	0 (0%)
		Metoprolol	6 (66.7%)	4 (66.7%)	2 (66.7%)
		Metoprolol and atenolol	1 (11.1%)	1 (16.7%)	0 (0%)
	Antidiabetics (insulin)		8 (6.9%)	4 (6.7%)	4 (7.1%)
	Loop diuretics/furosemide		3 (2.6%)	3 (5.0%)	0 (0%)
	Potassium sparing diuretics (spironolactone)		1 (0.9%)	1 (1.7%)	0 (0%)
	Miscellaneous medications	Antibiotics	44 (37.9%)	22 (36.7%)	22 (39.3%)
		Antiulcers	24 (20.7%)	13 (21.7%)	11 (19.6%)
		Anticonvulsants	10 (8.6%)	6 (10.0%)	4 (7.1%)
		Maintenance fluid (MF)	9 (7.8%)	5 (8.3%)	4 (7.1%)
		Anti-pains	6 (5.2%)	1 (1.7%)	5 (8.9%)
		Laxatives	6 (5.2%)	5 (8.3%)	1 (1.8%)
		others	14 (12.1%)	9 (15.0%)	5 (8.9%)
Other supportive (n = 115)	In-hospital rehabilitation therapy (bedside physiotherapy)		115 (99.1%)	60 (100%)	55(98.2%)
	Salt free diet		113 (97.4%)	59 (98.3%)	54 (96.4%)
	Sugar free diet		8 (6.4%)	4 (6.7%)	4 (7.1%)
Medications at discharge (n = 78)	Antihypertensive (n = 58)	ACEIS/ARBS	26 (44.8%)	17 (51.5%)	9(36.0%)
		Diuretics + ACEI	10 (17.2%)	7 (21.2%)	3(12.0%)
		Diuretics	9 (15.5%)	4(12.1%)	5 (20.0%)
		CCB	6 (10.3%)	2 (6.1%)	4 (16.0%)
		Diuretics + CCB	3 (5.2%)	0 (0%)	3 (12.0%)
		BB and ACEIs	2 (3.4%)	2 (6.1%)	0 (0%)
		CCB + ACEI	1 (1.7%)	0 (0%)	1 (4.0%)
		CCB + ACEIs + diuretics	1 (1.7%)	1 (3.0%)	0 (0%)
	Antiplatelet		49 (42.2%)	49 (81.7%)	0 (0%)
	Statins		49 (42.2%)	49 (81.7%)	0 (0%)
	Antibiotics		7 (6.0%)	3 (5.0%)	4 (7.1%)
	Antidiabetics		6 (5.2%)	3 (5.0%)	3 (5.4%)
	Anticoagulants		3 (2.6%)	3 (5.0%)	0 (0%)
	Others		10 (8.6%)	6 (10%)	4 (7.1%)

*ACEIs: angiotensin-converting enzyme inhibitors, ARBs: angiotensin II receptor blockers/antagonists, BB: beta blockers, CCB: Calcium channel blockers.

## 4 Discussion

Complications after stroke are common, present barriers to optimal recovery or related to poor outcomes and are potentially preventable or treatable [47]. During the rehabilitation process, patients are vulnerable to various complications as a result of both the stroke and the disability caused by it [27]. In-hospital medical complications are common and strongly associated with the risk of death and dependency among stroke patients [48]. In this study setup complications were prevalent in majority of the stroke patients (80.2%). This was comparable with observational study conducted at a tertiary care teaching hospital in Kadapa, 75% [47], the RANTTAS trial, 95% [23], study at university hospital of Trondheim in Norway, 82.4% [49], study by Civelek et al. conducted in Turkey, 88.9% [27] and prospective, multicenter study in the west of Scotland, 85% [50] of stroke patients had experienced at least one complication. But the rate of the complication was higher compared to a study conducted in ten Asian countries which revealed that 423 (41.9%) developed complications within the first 2 weeks of stroke [51] as well as study in Bromley hospitals NHS Trust, UK where medical complications were documented in 60% of stroke patients [52]. The higher rate complications among patients in our study might be due to severity of the disease itself, associated co-morbidities and set up of the hospital for the management of emergency chronic conditions due to shortage of appropriate laboratories, equipment's, medications and skilled man power.

Even though the severity of the complications varies in degrees due to the nature of the disease most patients might complained different complications as the study was prospective with face to face interview. Additionally, laboratory investigations were carefully interpreted to identify related complications. Thus, close monitoring of patients and detailed as well as timely note keeping may have contributed to the high rate of complications in this study. Improved assessment procedures and early rehabilitation resulting from standardized evaluation and early management protocols employed in previous study setup [51]. This variety of complications in different setups at different times might be due to difference in the study design, stroke subtype, co-morbidities and hospital service.

The most complication developed during the study period was brain edema (increased intracranial pressure) followed by urinary incontinence and aspiration pneumonia. These complications specifically brain edema and aspiration pneumonias were the common complications in consistent with other previous findings [23,37,38,[51], [52], [53], [54], [55]]. The most medical complication developed was urinary incontinence 33 (28.4%) which was similar to previous study findings [25,27,50].

Unlike our finding, urinary tract infection was the commonest and pneumonia was less compared to study by Deleu et al. in Arabian Gulf countries [56]. Aspiration pneumonia followed by urinary tract infection (UTI) were the most common complications developed as per study by Mamushet et al. in Ethiopia [55]. In this study electrolyte abnormalities were one of the major complications similar to study by Jia et al. in Fujian [53]. Brain edema and seizure attack were the most accountable complications identified in the prospective observational study at a tertiary hospital Andhra Pradesh, India [34]. Additionally recurrent stroke, chest infections and urinary tract infections were most commonly encountered as study by Navarro et al. [51]. Study at university hospital of Trondheim in Norway also indicated that the most common complication was pain (53.3%) followed by urinary tract infection in (27.9%) [49]. Neurological complications, such as brain edema or haemorrhagic transformation, occur earlier than do medical complications and can affect outcomes with potential serious short-term and long-term consequences [35].

Dysphagia is predominant after stroke and has been associated with an increased risk of pulmonary complications like aspiration pneumonia. Pneumonia remains as an important and a modifiable complication of stroke and measures to prevent such as reducing aspiration could definitely improves the overall outcome. Several studies performed with various designs in different centers revealed that the rate of complications after a stroke were varied. The results from these studies show a wide variation due to differences in the study designs, patient cohorts, selection and diagnostic criteria, stroke subtype, comorbidities, hospital service, LOS and duration of follow-up. Definition of complications is another factor that may contribute to differences in results across the studies.

Complications after stroke were common and major factors contributing to mortality. The severity of stroke on admission was the most important risk factor for developing complications. Complications are still frequent after stroke despite treatment in a comprehensive SU and close follow-up in a well-organized service [49]. Being aware of the types of common complications and associated risk factors helps the clinical team involved in the care of stroke patients to make preparations and plans for the best possible care and to take preventive measures [55].

The wide variation in hospital admission rates for stroke in developing countries is presumably a reflection of the resources available to the general practitioner, the proximity and quality of a hospital with an available bed and that is prepared to take stroke patients, expectations of the patient and family, local or national guidelines, and cost [30]. Quality-assurance audits linked to the severity of stroke, cardiovascular comorbidity, and length of stay should allow comparison of the spectrum and frequency of complications, need for acute-care hospital readmission, and mortality from one institution to another [36].The success of a stroke rehabilitation unit depends on the effective utilization of its resources and seamless coordination between different healthcare professionals as well as the ongoing support from the caregivers and other community services [24].

Areas open to improvement include stimulation of early health-seeking behavior, correct treatment of complications, adequate bed and home care, and compliance to treatment. Early control of nutrition and prevention of dehydration in cases of swallowing impairment are priorities. Infection prevention, prompt reaction to fever, and antibiotic treatment of infections, especially pneumonia, may significantly reduce mortality [26]. Individualized care plans should address nutrition, oral care, mobilization and incontinence, and reduce the risk of complications such as urinary tract infection, aspiration pneumonia, and venous thromboembolism [25]. This will save a lot of lives with best possible use of meager resources available such as educating the population to avoid oral feeding for patients with altered mental state and physicians to evaluate gag reflex bedside swallowing test and proper positioning of patients to avoid aspiration pneumonia [55]. Additionally, appropriate treatment of brain edema with drugs like mannitol and appropriate catheterization of the patient for urinary incontinence is necessary. The identification of such complications in the first days of effect helps to notice the early preventability with a high degree of functional outcome and can reduce the mortality or disability rate [34].

More than half of the patients had at least one past medication, mostly for the treatment of co-morbidities like diabetes mellitus, hypertension and heart failure. Antihypertensives were the commonly used prestroke medications as majority of the patients had previous history of hypertension. During hospitalization, majority of the patients (95.7%) had received at least one medication. Medications were given during hospitalization for the treatment of ischemic stroke, underlying conditions and commonly developed complications. Time to start of medication depends on the condition of the patient, type of stroke, imaging result, laboratory parameters and complications developed. During hospitalization the most common medications given for the patients were antiplatelets and statins mostly for ischemic stroke 63 (54.3%) patients. This complies with previous study as the most well studied treatments for ischemic stroke are antiplatelets and statins [18]. Study in Ethiopia by Temesgen et al. also reported that aspirin and statins were the most frequently used drugs in the management of stroke [41]. As a class, the most commonly prescribed drugs were antihypertensive, finding in line with other studies in which hypertension was the commonest risk factor for stroke [12, 42, 54].

In this study 7 patients with hemorrhagic stroke (6.0%) received antiplatelets wrongly before stroke was confirmed by imaging suspecting clinically to be ischemic stroke. This abnormality was due to the fact that brain imaging was malfunction or not done at all if patients or their attendants were not convinced of the benefit of the CT scan, or they were not willing or able to pay initially that delays the imaging. In these cases, physicians sometimes gave anticoagulants and antiplatelets if the patient was suspected of having ischemic stroke. When imaging result becomes available either through negotiation or when the CT scan function, these medications were immediately stopped if the patient is found to have hemorrhagic stroke contrary to the preliminary suspected diagnosis of ischemia. This highlights that despite the availability of the CT scan, educational level and economic capacity remain as major barriers to utilization of this important diagnostic tool among stroke patients. These patient management challenges, combined with inadequate rehabilitation services, lack of preventive measures, as well as poor understanding of the possible unique risk factors associated with stroke in LMICs, may account for the disproportionately large stroke burden borne by these countries [3].

The median time of start of antiplatelet after hospital arrival was 24 h that correlates with study by Bennour et al., where the mean was 1.2 ± 1.4 days [57]. Treatments were delayed until the diagnosis was confirmed by different physicians clinically or imaging along with consideration of different laboratory values. With this regard, 44 (37.9%) patients received antiplatelets within 24 h of hospital arrival. Heparin was the most anticoagulant drug prescribed mostly for prophylaxis and treatment of DVT and PE as well as for patients with atrial fibrillation. In some patients other anticoagulants like warfarin and enoxaparin as single or in combination were given. Unless DVT and PE are treated early, these conditions are the prominent causes of death. Patients having atrial fibrillation benefited from anticoagulants if they have high risk for the development of stroke.

During discharge about two third (67.2%) of the patients had received medication and anti-hypertensive were the prominent medications for discharge, given for half of the patients. At discharge, antiplatelet medication and lipid lowering medication were more commonly provided to patients with ischemic strokes than those with hemorrhagic strokes [18, 57]. This was consistent with our study that at discharge antiplatelets and statins were given for 49 (42.2%) patients with ischemic strokes alone.

Despite its effectiveness in improving neurological outcomes, many patients with ischemic stroke were not treated with r-tPA. In developing countries tPA and newly approved devices are unaffordable and there is inappropriate treatment due to misdiagnosis caused by lack of appropriate diagnostic agents [31]. The fact that the majority of our patients come late, creates management difficulties as these first hours are important to avoid secondary insults to the brain and preserve the ischemic penumbra. Considering the concept that “time is brain” these subset of patients should have the acute treatment option with IV r-tPA if available for them. Although thrombolytic treatment is currently not available in our country, significant delays during the pre-hospital or in-hospital phases, resource for management, diagnosis and lack of skilled man power creates management difficulties and would make such advanced treatments difficult in the future even if r-tPA is available in the local hospital.

Patients with a chronic condition like stroke may require lifelong pharmaceutical treatment, lifestyle maintenance and self-management skills, and caregiver and family support, in order to achieve optimal health outcomes. Rehabilitation improves physical, speech, and cognitive functioning of disabled stroke patients [6]. Low levels of specialist input and rehabilitation therapy likely reflect a lack of clinical practice guideline guidance or poor documentation, as well as limited resources and/or ineffective resource allocation. For example, during the study period in JUMC there was no neurologist and only a limited number of physicians were working in the stroke unit, presenting an impractical patient load. In parallel efforts should be made to establish best practices for acute stroke care in such settings.

### 4.1 Strength and limitations of the study

The major strength of this study was its prospective study design, the close observation of stroke patients after onset in a well-established and defined evidence-based stroke service as well as the enrollment of consecutive patients. This prospective study allowed collection of accurate data on sociodemographic, complications and treatments approaches. Hence, the study can inform stroke management strategies and interventions required to decrease mortality associated with stroke. The weakness of the study was first it was a hospital-based study rather than population based, hence may be subjected to referral bias. Additionally, the sample size was small hampering the analysis of some prognostic indicators due to the short recruitment period. Finally, although tissue plasminogen inhibitor is the standard of care, it was not given to any patient, because of unavailability and lack of standard protocol in our country.

## 5 Conclusion

Both neurologic and medical complications were common in majority of the patients, due to severity of the disease itself, associated comorbidities and set up of the hospital. The most complication developed during the study period was brain edema (increased intracranial pressure) followed by urinary incontinence and aspiration pneumonia. More than half of the patients had at least one past medication history and anti-hypertensive were the commonly used prestroke medications. During hospitalization majority of the patients had received at least one medication, from those about one third of them received within 3 h of hospital arrival. The most common medications prescribed during hospitalization were antiplatelets and statins mostly for ischemic stroke. During discharge about two third of the patients had received medication and anti-hypertensives were the prominent discharge medications prescribed for half of the total discharged patients. Despite the fact that stroke is treatable, quality care remains a mirage in SSA. In essence, most persons in SSA are not getting the stroke care they need: awareness, access and action are still very much below par. Additionally there was no r-tPA services for management of acute ischemic stroke patients.

Mitigating the mortality and degree of morbidity experienced by patients is dependent on rapid appropriate management from the time of symptom onset. It is therefore essential that patients be aware of the symptoms of stroke, as well as the importance of reporting immediately to a medical facility for evaluation and subsequent management. Creating a community education outreach team with public relations, volunteers, and hospital educators is necessary in minimizing current severity of stroke. Primary prevention, early diagnosis and appropriate management of stroke should be improved by education of the public and healthcare professionals.

Involvement of community and faith-based organizations, emphasis on examination to differentiate between hemorrhagic and ischemic stroke and training of community health professionals on essential management of stroke should be urgently implemented. Being aware of the types of common complications and associated risk factors, the clinical team involved in the care of stroke patients should make preparations and plans for the best possible care and to take preventive measures that will save a lot of lives. Emergency medical services (EMS) should be developed and upgraded for stroke care at the hospital or district level to include transport and triage of patients from peripheral medical centers.

We have recognized that in our hospital, the management protocol for stroke patients was sub-optimal and lagging behind the recommended guidelines due to lack of appropriate treatment and diagnostic agent for etiologic investigation in addition to trained man power. In addition, the ministry of health of the country should develop and implement generalized protocol guideline for in hospital management and post stroke follow-up. An expert panel should be convened to formulate consensus guidelines for the management of acute stroke of unknown etiology in settings where there is no rapid access to neuroimaging to determine the underlying etiology of stroke, as these settings account for a substantial proportion of the world's stroke patients. There is burning need to establish and strength the available stroke unit which are well-equipped and staffed intensive care units in different hospitals across the country for proper stroke care, to reduce stroke mortality and post stroke disability. We still have challenges and further research is needed regarding complications, as optimal prevention, management and effects on outcome have not been established for many complications.

## Ethical approval

Ethical clearance was obtained from the Institutional Review Board (IRB) of Jimma University, Institute of health with reference number of IHRPGC/107/207.

## Sources of funding

This work was funded by Jimma University. The funding body did not have any role in study design, data collection, data analysis, interpretation of data or in writing the manuscript.

## Author contribution

GF contributes in the proposal preparation, study design, analysis and write up the manuscript. LC, TM, BG and MG coordinated and led the research, oversaw design of the research protocol and questionnaires as well as provided comment to the draft manuscript. FB and GF assisted in drafting questionnaires, provided support for data collection and supervised the data collection. All authors read and approved the final version of the manuscript and agree to be accountable for all aspects of the work in ensuring that questions related to the accuracy or integrity of any part of the work are appropriately investigated and resolved.

## Trial registry number

1. Name of the registry: Research registry, https://www.researchregistry.com
2. Unique Identifying number or registration ID: researchregistry5112
3. Hyperlink to the registration (must be publicly accessible): https://www.researchregistry.com/register-now#home/registrationdetails/5d70f2520791fb0011b79e9f/

## Guarantor

Ginenus Fekadu.

## Consent

Not applicable. No individual person's personal details, images or videos are being used in this study.

## Provenance and peer review

Not commissioned, externally peer reviewed.

## Declaration of competing interest

The authors declared that they have no competing interest.
